# Book Notices

**Published:** 1890-09

**Authors:** 


### Book Notices.

RAILWAY SURGERY, a Practical Work on the Special Department of Railway Surgery; for Railway Surgeons and Practitioners in the General Practice of Surgery. By C. B. Stemen, A. M., M. D., LL. D., Professor of Surgery in the Fort Wayne College of Medicine, Indiana; Chief Surgeon of the Western Division of the Pittsburg, Fort Wayne and Chicago Railway, etc. With numerous illustrations. Pp. 306; 1890. J. H. Chambers & Co., St. Louis, Mo.

This seems to be the first book devoted exclusively to railway surgery that has been published. It is quite comprehensive and practical, and it deserves a place in every railway surgeon's library. It is estimated that there are about 25,000 surgeons connected, more or less, with railroads in this country. The first organization of railway surgeons was held in Decatur, Ill., in January, 1882. Since this time wonderful advances have been made along the line of railway surgery, and this book is the outcome of this special work. B.

---

ANÆSTHETICL, ANCIENT AND MODERN:—Their physiological action, therapeutic use, and mode of administration, together with an historical resume of the introduction of modern anæsthetics. Nitrous oxide, ether, chloroform and cocaine; and also an account of the more celebrated anæsthetics in use from the earliest time to the discovery of nitrous oxide, by Geo. Foy, F. R. C. S.; fellow of the Royal Academy of Medicine in Ireland; Surgeon to the Whitworth Hospital, Drumcondra, etc., London. Bailliere, Tindall & Co., Dublin. Fannin & Co., Grafton street, 1889, pp. 175.

This little book is dedicated to Hunter McGuire, M. D., LL.D., of Richmond, Va., and covers the field of historical and practical anæsthetics. Any one wishing to be well informed on the subject will find this a good book from which to obtain information. Too much of the book is taken up with the advertisements of the publishers' publications to look well. B.

---

MANUAL OF SKIN DISEASES with special reference to diagnosis and treatment. For the use of students and general practitioners. By W. A. Hardaway, M. D., professor of skin diseases in the Missouri Medical College, and in the St. Louis Post-Graduate School of Medicine, etc., St. Louis. Theo. F. Lange, 1890, pp. 530. Price, cloth $3.

This book is really a valuable production. The long monotonous generalizations are properly left out. The work is a practical one. The author emphasizes the diagnosis and treatment. The first part of the book is devoted to a short introduction to the study of skin diseases, the second, to an alphabetical arrangement of diseases for ready reference, and the third and concluding portion to formulæ and a diet table. B.

---

A HAND-BOOK OF PATHOLOGICAL ANATOMY AND HISTOLOGY, with an Introductory Section on *post mortem examinations*, and the methods of preserving and examining *diseased tissues*. By Francis Delafield, M. D., Professor of Pathology and Practical Medicine, College of Physicians and Surgeons, New York; and T. Mitchell Prudden, M. D., Director of the Laboratory of the Alumni Association of the College of Physicians and Surgeons, New York. Third edition. Illustrated by 224 wood engravings, printed in black and colors. 560 pp. New York: Wm. Wood & Co.; 1890.

This work has enjoyed the distinction of being a standard work, and it having run through three editions evinces its popularity. The prudent physician will not allow a book of this kind to escape his library. The student and practitioner are informed how to perform autopsies; to preserve the tissues, to prepare them properly and to examine them with the microscope. All of the recent advances in pathology and histology are set forth in good style. B.

---

MANUAL NURSING:—By A. Worcester, A. M., M. D., Fellow of the Massachusetts Medical Society, Physician to the Watham Hospital, etc. Second edition, pp. 250, New York. D. Appleton & Co., 1890.

The subject of nursing is receiving more attention and is more encouraged by the profession than at any time in the past. A well trained nurse is invaluable in the sick room. This little book nstructs in the art of monthly nursing. The contents are substance of lectures delivered to the nurses at the Boston Lying-in Hospital during the author's term of service as house physician in 1883.

The book is well written, well printed and contains the best of information on the subject of monthly nursing.

SPINAL CONCUSSION, Surgically considered as a cause of Spinal Injury, and neurologically restricted to a certain symptom group, for which is suggested the designation Erichsen's Disease, as one form of the traumatic neuroses, by S. V. Clevenger, M. D., Chicago, with thirty-two wood engravings, Philadelphia. F. A. Davis, Publisher, 1889. Price, $2.50.

Dr. Clevenger has given this subject special study for five years, and the outcome has been a splendid work. He has reviewed the entire literature of spinal concussion and made many additions himself. His long experience as a scientific student, writer and teacher, has well qualified him for the work set forth in this volume.

The book contains thirteen chapters in which are given the history, recent advances, symptoms, diagnosis, pathology, treatment, the last chapter being devoted to medico-legal considerations. It is a work for the lawyer as well as for the physician.

